# *ACE* I/D Genotype and Risk of Non-Contact Injury in Moroccan Elite Athletes: A Pilot Study

**DOI:** 10.3390/medicina61010098

**Published:** 2025-01-10

**Authors:** El Mokhtar El Ouali, Jihan Kartibou, Juan Del Coso, Rashmi Supriya, Ismail Laher, Zineb El Kettani, Hassan Ghazal, Najib Al Idrissi, Ayoub Saeidi, Abdelhalem Mesfioui, Hassane Zouhal

**Affiliations:** 1 Institute of Sports Sciences, Hassan I University, Settat 26002, Morocco; elmokhtar.elouali@uit.ac.ma; 2 Laboratory of Biology and Health, Department of Biology, Ibn Tofail University of Kenitra, Kenitra 14000, Morocco; jihan.kartibou@usmba.ac.ma (J.K.); a.mesfioui@yahoo.fr (A.M.); 3 Sport Sciences Research Centre, Rey Juan Carlos University, 28942 Fuenlabrada, Spain; juan.delcoso@urjc.es; 4 Centre for Health and Exercise Science Research, Hong Kong Baptist University, Kowloon Tong, Hong Kong 999077, China; 5 Department of Sport, Physical Education and Health, Academy of Wellness and Human Development, Faculty of Arts and Social Sciences, Hong Kong Baptist University, Kowloon Tong, Hong Kong 999077, China; 6 Department of Anesthesiology, Pharmacology and Therapeutics, The University of British Columbia, Vancouver, BC V6T 1Z4, Canada; ismail.laher@ubc.ca; 7 Laboratory of Genomics, Epigenetics, Bioinformatics, Personalized and Predictive Medicine, Mohammed VI University of Sciences and Health, Casablanca 20000, Morocco; zineb.elket5@gmail.com (Z.E.K.); hassan.ghazal@fulbright.org (H.G.); nalidrissi@um6ss.ma (N.A.I.); 8 Institut Royal de la Formation des Cadres pour la Jeunesse et le Sport, Salé 10000, Morocco; 9 Department of Physical Education and Sport Sciences, Faculty of Humanities and Social Sciences, University of Kurdistan, Sanandaj 1517566177, Iran; 10 M2S (Laboratoire Mouvement, Sport, Santé)—EA 1274, University Rennes, 35000 Rennes, France; 11 Institut International des Sciences du Sport (2I2S), 35850 Irodouer, France

**Keywords:** angiotensin-converting enzyme, genotypes, endurance athletes, team sports athletes, tissue damage, athletic performance

## Abstract

*Background and Objectives:* The insertion/deletion (I/D) polymorphism in *ACE*, the gene encoding the angiotensin-converting enzyme (ACE), has been suggested as a genetic variation that can influence exercise performance and risk of injury in elite athletes. The I allele has been associated with enhanced endurance performance and with reduced inflammation, while the D allele has been associated with improved performance in strength and power activities. However, the role of this genetic variant in the incidence of non-contact injury is underexplored. This study investigated the possible association of *ACE* I/D genotypes with the risk of non-contact injury in elite Moroccan athletes. *Materials and Methods:* Forty-three elite male athletes (19 cyclists and 24 field hockey players) from the Moroccan national team participated voluntarily. Non-contact injuries were recorded for all athletes and classified according to the IOC consensus statement by the medical staff of the teams. *ACE* I/D polymorphism genotyping was performed by polymerase chain reaction (PCR) using genomic DNA from blood samples. *Results:* There were four cyclists (21.05%) and eight field hockey players (33.33%) with a non-contact injury during the season. The distribution of the *ACE* I/D genotypes was similar in the athletes with vs. without non-contact injury for cyclists (DD/ID/II 25.00/50.00/25.00% vs. 46.67/40.00/13.33% non-injured, respectively; X^2^ = 0.69, *p* = 0.70), field hockey players (DD/ID/II 50.00/50.00/0.00% vs. 50.00/43.75/6.25%; X^2^ = 0.54, *p* = 0.76) and for the whole group of athletes (DD/ID/II 41.67/50.00/8.33% vs. 48.39/41.94/9.68%; X^2^ = 0.22, *p* = 0.89). In the whole group of athletes, neither the dominant (DD + ID vs. II = OR: 1.17, 95% CI: 0.15–16.56, *p* = 0.89) nor the recessive (DD vs. ID + II = OR: 1.31, 95% CI: 1.31–4.89, *p* = 0.69) models showed an increased risk of non-contact injury. *Conclusions:* The distribution of the *ACE* I/D genotypes was similar in elite cycling and field hockey athletes with or without non-contact injury during the season. These results indicate that there is no significant association between the *ACE* I/D polymorphism and the susceptibility to non-contact injury in these athletes. Further research is warranted to validate these findings and to investigate their broader implications for advancing knowledge in sports injury prevention and optimizing athlete management strategies.

## 1. Introduction

The relentless accumulation of physical and physiological stress related to professional sports, coupled with limited recovery intervals after training sessions or competitions, predisposes athletes to non-contact injury, which is defined as tissue damage that occurs without direct external trauma [1]. Non-contact injury is generally the result of a failure of body tissues to resist the stress imposed by habitual locomotion and can be induced by over-exertion that produces structural tissue damage [2,3]. Generally, non-contact injury includes most types of body tissues, such as ligaments [4], bones [5], tendons [6], nerves [7] and muscles [8], although muscle-type injury is the most common type of non-contact injury in sport [9]. Overall, around 92% of non-contact muscle injuries affect the four main muscle groups of the lower limbs, with the hamstrings being the most frequently affected (37%), followed by the adductors (23%), quadriceps (19%) and calf muscles (13%) [1]. A number of intrinsic and extrinsic factors can influence injury incidence and severity in athletes, including performance level, training approach, recovery, diet and inter-individual variability [10,11,12]. Previous studies have suggested that genetic factors may also be a determining factor in susceptibility to non-contact injury [11,13,14].

Various genetic polymorphisms have been associated with an increased risk of non-contact injury in elite athletes [15,16,17,18]. The insertion/deletion (I/D) polymorphism of the *ACE* gene is an important genetic variant, as this gene encodes the angiotensin-converting enzyme (ACE), a protein that mediates several process of muscle growth, blood flow regulation and mechanical efficiency [19,20]. Specifically, ACE is a key enzymatic component of the renin–angiotensin system (RAS), as it enables faster conversion of angiotensin I (Ang I) to angiotensin II (Ang II). A higher ACE activity in bodily tissues, mainly in the blood stream, promotes vasoconstriction [21,22]. Blood concentrations of ACE can be affected by I/D polymorphisms of the *ACE* gene [23,24]. Individuals homozygous for the I allele (i.e., II genotype) have reduced serum and tissue ACE activity and exhibit less vasoconstriction with increased blood flow to muscles during mechanical work [25,26]. In contrast, carriers of the D-allele polymorphisms (DD and ID genotypes) have higher levels of serum and tissue ACE activities, resulting in greater vasoconstriction and a higher proportion of type 2X fast muscle fibers [27]. The *ACE* gene has been identified as the most important candidate gene for athletic performance, as the II genotype may be overrepresented in endurance athletes, while the DD genotype is overrepresented in power-sports athletes [28]. These data suggest that there may be benefits of possessing the II genotype for endurance performance as a consequence of lower vasoconstriction and benefits of possessing the DD genotype for power-based performance due to a higher proportion of fast-type muscle fibers.

Several investigations have reported an association of the *ACE* I/D polymorphism with various exercise-related phenotypes; these correlations encompass disparities in the distribution and composition of skeletal muscle fibers [27], as well as improvements in capillarization [29]. Additionally, this genetic variation may impact muscle metabolism [30], muscle strength [31], muscle volume [32] and the ability to resist fatigue after exercise training [33]. Beyond the phenotypic effects of the *ACE* I/D polymorphism, the influence of this genetic variant on exercise performance could also be attributed to a higher susceptibility to non-contact injuries [34]. According to Yamin et al. [35], there were differences in the concentration of serum creatine kinase (CK, a marker of muscle damage) in individuals with different *ACE* I/D genotypes after eccentric exercise. Specifically, DD individuals had lower peak CK values than those with the II genotype after exercise. Additionally, reduced CK concentrations were measured after triathlon [36] and marathon competitions [37] in participants with the D allele. Consistent with this finding, endurance runners with the D allele also had reduced inflammation and muscle injury after a marathon [38]. Collectively, these findings suggest that the D allele may provide phenotypic advantages by enhancing resistance to muscle strain during intense exercise, potentially reducing the susceptibility to non-contact injuries, particularly muscle-related injuries, in athletes with DD and ID genotypes. This hypothesis was recently confirmed in football players, where the DD and ID genotypes were found to be underrepresented in players with muscle injuries compared to those without muscle injuries [17]. However, this hypothesis has not been tested in other populations of elite athletes. The aim of this study was to investigate the possible association of the *ACE* I/D genotypes with the risk of non-contact injury in elite cyclists and field hockey players. We hypothesized that athletes with the DD and ID genotypes would be underrepresented in the group of athletes with non-contact injury relative to their counterparts without injury, as they possess phenotypes that reduce their susceptibility to injury.

## 2. Materials and Methods

### 2.1. Ethics Statement

This study was conducted following the principles of the Declaration of Helsinki and approved by the Institutional Review Board of the Doctoral Center of Ibn Tofail University (ethics code: 23-2020; approval date: 24 September 2020). A full description of the processes involved, as well as possible risks and benefits related to this study, was provided to all participants. Participants voluntarily signed an informed consent form indicating their agreement to participate in the intervention. The procedures complied with the International Federation of Sports Medicine consensus statement on genetic information [39]. All athletes received an individual report with information about their *ACE* genotype as compensation for their participation in this study (after completion of the study).

### 2.2. Study Design and Participants

Forty-three Moroccan elite male athletes participated voluntarily in this study. Of this sample, there were 19 cyclists representing the Moroccan national cycling team, of which two athletes qualified for the Paris 2024 Olympic Games, six qualified for the 2023 World Championships and two already won the Tours of Africa. The remaining 24 athletes were part of the Moroccan national field hockey team, which ranked sixth in Africa and forty-ninth in the world according to the latest continental and international rankings. All participants underwent anthropometric measurements under uniform conditions, and 4 mL blood samples were collected by trained nurses according to standard safety protocols. An alphanumeric code was assigned to each tube of blood to ensure confidentiality. The tubes were then stored at −80 °C for later analysis.

### 2.3. Injury Collection

Data on the details of non-contact injury were obtained from all athletes prospectively. Sports-related injuries were diagnosed, classified and recorded by the medical staff of the Moroccan national cycling and field hockey federations using the classification system developed by the medical commission of the International Olympic Committee (IOC) [40] and its extension for cycling [41]. The IOC consensus statement on injury recording provides standardized guidelines for recording and reporting injuries and illnesses in sport. Key components include clear criteria for defining injuries and categorizing severity, standardized forms to assess athlete exposure, mechanisms of injury and diagnostic details and the use of internationally recognized systems (e.g., Orchard Sports Injury Classification System) for consistent categorization of injury types and locations. Briefly, non-contact injuries were recorded during the 2022/2023 sports season for all athletes (September to July for cycling and August to June for field hockey). We focused exclusively on non-contact tissue injury that occurred during training sessions or competitions and that were diagnosed by medical experts. Injuries caused due to collision with another cyclist/player or with an object (either direct or indirect) were excluded from the investigation as they were likely unrelated to the player’s genotype. The medical staff of the teams used an ad hoc questionnaire developed by the research team to record each injury. This questionnaire was completed following each non-contact injury during the sports season and was sent to the researchers via email. The sample of this study was divided into two groups according to the aims of this investigation: (a) injury group, related to all athletes that suffered at least one non-contact tissue injury during the season, and (b) non-injury group, related to the remaining athletes with no reports of non-contact injury over the season.

### 2.4. Genotyping

Genomic deoxyribonucleic acid (DNA) extraction was carried out from leukocyte samples using a commercial kit (MagPurix Blood DNA Extraction Kit, New Taipei City, Taiwan) following the manufacturer’s instructions. DNA concentrations were assessed by a nanodrop test using a microspectrophotometer. Genotyping was determined by polymerase chain reaction (PCR) using a thermal cycler, including the VERITYTM instrument from Applied Biosystems. The design of the primers and the experimental protocol have already been described [42]: forward primer: 5′-CTGGAGACCACTCCCATCCTTTCT-3′ and reverse primer: 5′-GATGTGG CCATCACATTCGTCAGAT-3′. The PCR protocol began with an initial denaturation step at 98 °C for 120 s, followed by 30 cycles of amplification, with each cycle including denaturation at 98 °C for 20 s, annealing at 58 °C for 60 s and extension at 72 °C for 30 s. After the amplification cycles, a final extension step was performed at 72 °C for a total of 180 s. The resulting PCR products were then electrophoresed on a 1% agarose gel with a 1 kb molecular weight marker for easy visualization. PCR genotyping of *ACE* I/D polymorphism was determined according to protocols described previously by others [43]. The *ACE* I/D polymorphism was based on the length of the fragments resulting from amplification with the primers mentioned above. The I allele was identified by the presence of a 490 base pair fragment, while the D allele was marked by the presence of a 190 base pair fragment. Specifically, the I/I genotype of the *ACE* gene was determined by the presence of a single 490 base pair band, indicating a homozygous insertion polymorphism. On the other hand, the I/D genotype is distinguished by the presence of two bands, one at 490 base pairs and the other at 190 base pairs. This pattern indicates a heterozygous state, i.e., the presence of insertion and deletion polymorphisms. Finally, the D/D genotype was recognized by a single band of 190 base pairs, indicating a homozygous deletion polymorphism. The laboratory provided genotypic identifications for each alphanumeric code or sample. The researchers were then able to correlate this anonymized genetic information with the respective athletes. This approach was used to protect the confidentiality and privacy of the athletes, ensuring that their identity was not disclosed throughout the genotyping process.

### 2.5. Statistical Analysis

A chi-squared (χ^2^) test was used to assess whether the samples conformed to the genotypic frequencies of the Hardy–Weinberg equilibrium (HWE). This assessment consisted in comparing the observed genotype frequencies within each group with the expected genotype frequencies based on the principles of Hardy–Weinberg equilibrium. To analyze categorical variables and compare the genotype frequencies across all groups, chi-squared (χ^2^) and Fisher’s exact tests were used. Furthermore, for comparisons between injury and non-injury groups in terms of genotypes (DD, ID and II) in the cycling, field hockey and all athletes’ groups, χ^2^ was used. Additionally, when the expected cell number was low, we used Fisher’s exact test, which is more suitable for handling low-frequency data. For the dominant (DD + ID vs. II) and recessive (DD vs. ID + II) models in the injury and non-injury groups, we used χ^2^ tests and calculated odds ratios (ORs) with 95% confidence intervals (CIs) to determine the influence of these models to the susceptibility of non-contact injury. Concerning continuous variables (anthropometric data), visual inspection (QQ-Plots) and the D’Agostino–Pearson test were used to assess normality. For comparison between groups of anthropometric data, we used one-way analysis of variance (ANOVA) when the data had a normal distribution and the Kruskal–Wallis test when the data did not follow a normal distribution. Data are presented as means and standard deviations (SDs) for normally distributed data and medians and interquartile ranges (IQRs, Q1–Q3) for data that were not normally distributed. The statistical significance threshold was set at *p* < 0.05 for all analyses. Statistical analysis was performed using GraphPad Prism 9.2.0 (GraphPad Software Inc., San Diego, CA, USA).

## 3. Results

From the total sample of 43 participating athletes, 12 athletes suffered a non-contact injury over the season (injury group; 4 cyclists and 8 field hockey players), while the remaining 31 athletes had no non-contact injuries (non-injury group; 15 cyclists and 16 field hockey players). There were significant differences in the anthropometric measurements between the injury and non-injury groups (*p* > 0.05; Table 1).

**Figure 1:**
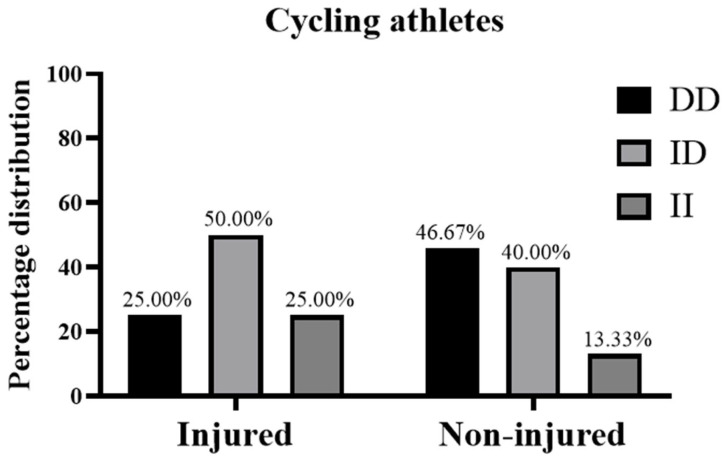
Genotype frequencies of *ACE* I/D polymorphisms in cyclists with non-contact injury during the 2022–2023 season and counterparts with no injuries during the same season.

**Figure 2:**
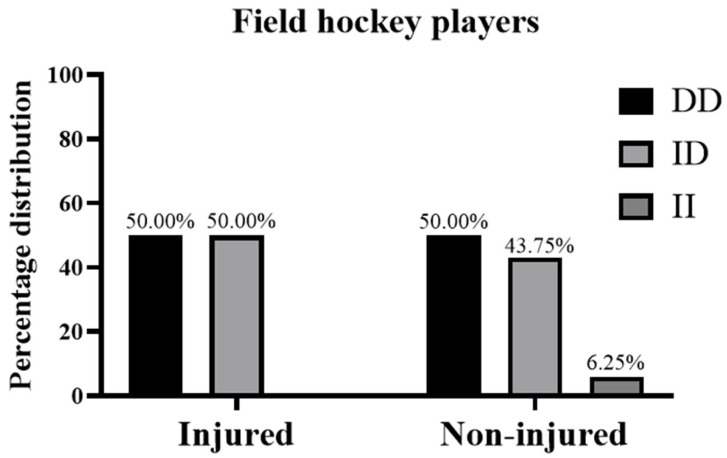
Genotype frequencies of *ACE* I/D polymorphisms in field hockey players with non-contact injuries during the 2022–2023 season and counterparts with no injuries during the same season.

There was a 100% success rate of genotype determination, and the distribution of *ACE* genotypes in all athletes did not deviate significantly from HWE (*p* = 0.98). The distribution of *ACE* I/D genotypes in the injury and non-injury groups was the following: in cyclists, the *ACE* genotype as DD/ID/II was 25.0/50.0/25.0% in the injury group and 46.7/40.0/13.3% in the non-injury group (Figure 1); in field hockey players, the *ACE* genotype as DD/ID/II was 50.0/50.0/0.0% in the injury group and 50.0/43.8/6.2% in the non-injury group (Figure 2).

**Figure 3:**
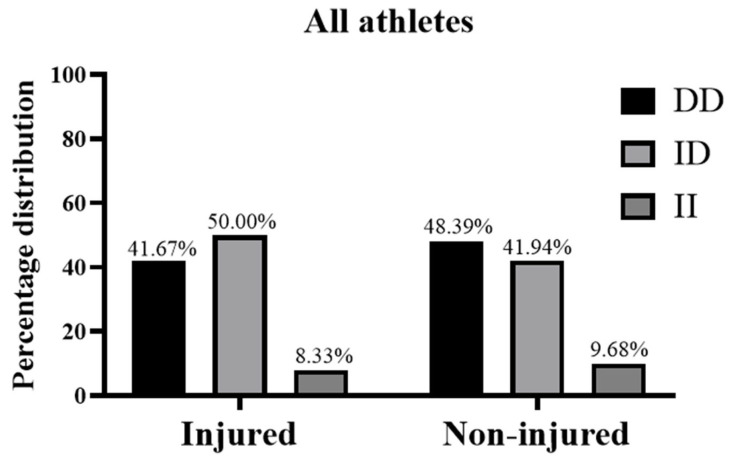
Genotype frequencies of *ACE* I/D polymorphisms in all athletes with non-contact injury during the 2022–2023 season and counterparts with injuries during the same season.

Finally, related to the frequency of *ACE* genotypes in the whole group of athletes, the *ACE* genotype (as DD/ID/II) was 41.7/50.0/9.7% in the injury group and 48.4/41.9/9.7% in the non-injury group (Figure 3).

There were no significant differences in the distribution of the *ACE* genotypes between the injury and non-injury groups for cyclists (χ^2^ = 0.69, *p* = 0.70), field hockey players (χ^2^ = 0.54, *p* = 0.76) and all athletes (χ^2^ = 0.22, *p* = 0.89). Furthermore, the dominant model of the *ACE* I/D polymorphism (DD + ID vs. II) did not indicate a higher risk of non-contact injury in cyclists (OR: 0.46, CI 95%: 0.04–8.74, *p* = 0.57), field hockey players (OR: infinity, 95% CI: 0.05-infinity, *p* = 0.47) and the whole group of athletes (OR: 1.17, 95% CI: 0.15–16.56, *p* = 0.89). Similarly, the recessive model (DD vs. ID + II) did not indicate any effect on the risk of non-contact injury in cyclists (OR: 2.62, 95% CI: 0.30–38.34, *p* = 0.43), field hockey players (OR: 1.00, 95% CI: 0.21–4.71, *p* > 0.999) and the whole group of athletes (OR: 1.31, 95% CI: 1.31–4.89, *p* = 0.69; Table 2).

## 4. Discussion

The goal of our study was to investigate the potential link between *ACE* I/D polymorphisms and the occurrence of non-contact injury in elite Moroccan cyclists and field hockey player. We performed this analysis based on previous investigations suggesting that the genetic pool in general [44], and specifically the I/D polymorphism of the *ACE* gene [17], may affect the risk of non-contact injury in elite athletes. Previous investigations reported differential phenotypes associated with the *ACE* I/D genotypes that could support the physiological mechanism(s) for increased risk of injury in II athletes with respect to those carrying the D allele (DD and ID) [25,26,27,31,32,33,34,35,36,37,38]. Specifically, individuals with II genotypes have reduced serum and tissue ACE [25,26] which contributes to a lower proportion of fast-type muscle fibers [27], lower muscle strength [31] and muscle volume [32] and higher levels of muscle damage and inflammation after several kinds of exercise [35] than D-allele carriers. However, our results do not confirm this hypothesis in cyclists and hockey players, as there were no differences in the distribution of *ACE* I/D genotypes between athletes that suffered a non-contact injury and those without injury over the same competitive season. Interestingly, the lack of differences in the distribution of the *ACE* I/D genotypes was also present for the whole group of athletes and for the subgroups of cyclists and field hockey players, suggesting that there is a lack of influence of this gene on the risk of injury either in endurance or team sports. Lastly, the dominant (DD + ID vs. II) and recessive (DD vs. ID + II) models of the *ACE* I/D polymorphism did not modify the risk of injury in this group of athletes. Collectively, these results indicate no significant association between *ACE* I/D polymorphisms and susceptibility to non-contact injury in a sample of elite Moroccan cycling and field hockey athletes.

Other studies also reported no association between the *ACE* I/D polymorphism and the incidence of non-contact injury in elite endurance football players [45], elite endurance athletes [46] and rugby players [47]. In contrast, a study by Massida et al. [17] reported an association between the D allele of the *ACE* I/D polymorphism and the prevalence of non-contact injury. Specifically, in their study of a Japanese cohort of professional football players, they found that players with the D allele had an odds ratio of 0.48 to suffer a non-contact injury compared to those with the II genotype, suggesting a protective effect of the D allele. The same study validated their findings with a meta-analysis of data collected from an Italian cohort of professional football players. This meta-analysis indicated that the frequency of the D allele was lower in the group of injured players than in the group of non-injured players, so that possessing the D allele reduced the risk of muscle injury by 31% [17]. The protective role of the D allele against injury has been also confirmed in Brazilian professional football players [48]. Based on the available studies, it is difficult to determine whether the *ACE* I/D polymorphism influences the risk of suffering a non-contact injury, as different outcomes have been used in studies investigating the same type of injury (non-contact injury) in athletes at the same level (international/elite/professional) and sports (endurance and team sports).

Individuals with the *ACE* D allele have an increased production of angiotensin II and a reduced half-life of bradykinin [17]. Angiotensin II and bradykinin are involved in inflammation due to tissue damage [34], implying that protection against tissue damage may be associated with higher levels of ACE activity [43], which is characteristic of D-allele individuals. Despite the evidence supporting a potential protective role of the D allele in the *ACE* gene (II genotype), our investigation did not find an effect of this genotype in elite cyclists and field hockey players. It is likely that the greater potential risk of injury due to genetic factors may be compensated by other modifiable factors such as the management of training and competition load to avoid excessive fatigue, stretching exercise to assure correct range of motion and the maintenance of appropriate nutrition guidelines [49,50]. Consequently, understanding the potential effects of the *ACE* I/D polymorphism and other genetic biomarkers, in conjunction with training, nutrition and environmental factors, could help optimize injury prevention strategies in elite athletes.

## 5. Limitations

Although our study is the first to examine the association between the *ACE* gene and the risk of non-contact injury in elite cycling and field hockey athletes, it is not without limitations. In fact, our analysis did not take into account the volume and intensity of training, competitions, diet habits or environmental conditions, which may have provided a better understanding of the link between external load over the season, the development of non-contact injury and the potential genetic predisposition. In addition, the number of athletes participating in this study was relatively limited (n = 43), which may reduce the statistical power. Furthermore, our study focused exclusively on elite male athletes, and it is possible that the effect of the variants in the *ACE* gene on non-contact injury risk differs in female athletes or in male athletes of a lower performance level. Thus, this analysis should be considered a pilot study. Further studies that combine assessment of injury risk in athletes with the assessment of *ACE* genotypes in combination with other polymorphisms such as the R577X variant of the ACTN3 gene would be useful, as the potential protective effect of the *ACE* D allele on the risk of non-contact injury may need the presence of the ACTN3 R allele [48]. Additionally, our study focused on a single polymorphism; however, it is important to recognize that tissue damage during training and competition is a multifactorial phenomenon, and its origins may involve various genes, polymorphisms, environmental factors and their complex interactions [51,52,53].

## 6. Conclusions

In summary, our results indicate no significant difference in the frequencies of genotypes (DD, ID and II) of the *ACE* I/D polymorphism between elite Moroccan cycling and field hockey athletes who sustained non-contact injuries and those without any non-contact injuries during the same season. This is contrary to our initial hypothesis, as we hypothesized that athletes with the DD and ID genotypes would be underrepresented in the group of athletes with non-contact injury. Collectively, our study reports no association between the *ACE* I/D polymorphism and susceptibility to non-contact injury in elite cycling and field hockey athletes. As a practical application, the data of this study suggest that genetic profiling based on the *ACE* I/D polymorphism may not be a reliable tool for predicting non-contact injury risk in elite cycling and field hockey athletes. Instead, coaches and sports medicine professionals should focus on modifiable risk factors, such as biomechanics, training load, recovery protocols and muscle conditioning, to prevent non-contact injuries.

## Figures and Tables

**Table 1:** Age and anthropometric variables of an elite group of Moroccan athletes with a non-contact injury during the 2022–2023 season and their counterparts with no injuries during the same season.

	All Athletes	Injury	No Injury	*p*-Value
Age (yr)	20 (18–23)	21.83 ± 3.86	20 (18–23)	0.80
Weight (kg)	64.30 ± 6.23	65.83 ± 4.59	64 (60–67)	0.22
Height (m)	1.77 ± 0.05	1.78 ± 0.04	1.76 ± 0.05	0.65
BMI (kg/m^2^)	20.64 ± 1.93	20.87 ± 1.43	20.55 ± 2.11	0.89

BMI = body mass index.

**Table 2:** *ACE* I/D genotypes in elite Moroccan athletes (cyclists and field hockey players) with non-contact injury during the 2022–2023 season and counterparts with no injuries during the same season.

		N (%)					Dominant (DD + ID vs. II)		Recessive (DD vs. ID + II)	
Groups	Genotype	Injury	Non-Injury	χ^2^	df	*p*-Value	OR [95% CI]	*p*-Value	OR [95% CI]	*p*-Value
Cyclists	DD	1 (25.00%)	7 (46.67%)	0.69	2	0.7	0.46 [0.04–8.74]	0.57	2.62 [0.30–38.34]	0.43
	ID	2 (50.00%)	6 (40.00%)							
	II	1 (25.00%)	2 (13.33%)							
Field hockey players	DD	4 (50.00%)	8 (50.00%)	0.54	2	0.76	Infinity [0.05–infinity]	0.47	1.00 [0.21–4.71]	>0.999
	ID	4 (50.00%)	7 (43.75%)							
	II	0 (0%)	1 (6.25%)							
All athletes	DD	5 (41.67%)	15 (48.39%)	0.22	2	0.89	1.17 [0.15–16.56]	0.89	1.31 [1.31–4.89]	0.69
	ID	6 (50.00%)	13 (41.94%)							
	II	1 (8.33%)	3 (9.68%)							

df: degree of freedom.

## Data Availability

The raw data supporting the conclusions of this article will be made available by the authors upon request.

## References

[1] Ekstrand J., Hägglund M., Waldén M. (2011). Epidemiology of Muscle Injuries in Professional Football (Soccer). Am. J. Sports Med..

[2] Delecroix B., McCall A., Dawson B., Berthoin S., Dupont G. (2018). Workload and Non-Contact Injury Incidence in Elite Football Players Competing in European Leagues. Eur. J. Sport Sci..

[3] McCall A., Dupont G., Ekstrand J. (2018). Internal Workload and Non-Contact Injury: A One-Season Study of Five Teams from the UEFA Elite Club Injury Study. Br. J. Sports Med..

[4] Chia L., De Oliveira Silva D., Whalan M., McKay M.J., Sullivan J., Fuller C.W., Pappas E. (2022). Non-Contact Anterior Cruciate Ligament Injury Epidemiology in Team-Ball Sports: A Systematic Review with Meta-Analysis by Sex, Age, Sport, Participation Level, and Exposure Type. Sports Med..

[5] Sonkodi B., Bardoni R., Poór G. (2022). Osteoporosis in Light of a New Mechanism Theory of Delayed Onset Muscle Soreness and Non-Contact Anterior Cruciate Ligament Injury. Int. J. Mol. Sci..

[6] Khoshnoodi P., Tehranzadeh A.D., Dunn J.M., Tehranzadeh J. (2014). Semimembranosus Tendon Avulsion Fracture of the Posteromedial Tibial Plateau Associated with Posterior Cruciate Ligament Tear and Capsular Rupture. Skelet. Radiol..

[7] Prien A., Feddermann-Demont N., Verhagen E., Twisk J., Junge A. (2020). Neurocognitive Performance and Mental Health of Retired Female Football Players Compared to Non-Contact Sport Athletes. BMJ Open Sport Exerc. Med..

[8] Bezuglov E., Talibov O., Butovskiy M., Lyubushkina A., Khaitin V., Lazarev A., Achkasov E., Waśkiewicz Z., Rosemann T., Nikolaidis P.T. (2020). The Prevalence of Non-Contact Muscle Injuries of the Lower Limb in Professional Soccer Players Who Perform Salah Regularly: A Retrospective Cohort Study. J. Orthop. Surg. Res..

[9] Edouard P., Navarro L., Branco P., Gremeaux V., Timpka T., Junge A. (2020). Injury Frequency and Characteristics (Location, Type, Cause and Severity) Differed Significantly among Athletics (‘track and Field’) Disciplines during 14 International Championships (2007–2018): Implications for Medical Service Planning. Br. J. Sports Med..

[10] Myerson S., Hemingway H., Budget R., Martin J., Humphries S., Montgomery H. (1999). Human Angiotensin I-Converting Enzyme Gene and Endurance Performance. J. Appl. Physiol..

[11] Lindpaintner K., Pfeffer M.A., Kreutz R., Stampfer M.J., Grodstein F., LaMotte F., Buring J., Hennekens C.H. (1995). A Prospective Evaluation of an Angiotensin-Converting-Enzyme Gene Polymorphism and the Risk of Ischemic Heart Disease. N. Engl. J. Med..

[12] Jones N., Kiely J., Suraci B., Collins D.J., de Lorenzo D., Pickering C., Grimaldi K.A. (2016). A Genetic-Based Algorithm for Personalized Resistance Training. Biol. Sport.

[13] Ahmetov I.I., Hall E.C.R., Semenova E.A., Pranckevičienė E., Ginevičienė V., Makowski G.S. (2022). Chapter Five—Advances in Sports Genomics. Advances in Clinical Chemistry.

[14] McCall A., Carling C., Nedelec M., Davison M., Le Gall F., Berthoin S., Dupont G. (2014). Risk Factors, Testing and Preventative Strategies for Non-Contact Injuries in Professional Football: Current Perceptions and Practices of 44 Teams from Various Premier Leagues. Br. J. Sports Med..

[15] Coelho D.B., Pimenta E.M., Rosse I.C., Veneroso C., Pussieldi G.D.A., Becker L.K., Oliveira E.C., Carvalho M.R.S., Silami-Garcia E. (2019). Alpha-Actinin-3 R577x Polymorphism Influences Muscle Damage and Hormonal Responses After a Soccer Game. J. Strength Cond. Res..

[16] Massidda M., Bachis V., Corrias L., Piras F., Scorcu M., Culigioni C., Masala D., Calò C.M. (2015). ACTN3 R577X Polymorphism Is Not Associated with Team Sport Athletic Status in Italians. Sports Med. Open.

[17] Massidda M., Myamoto-Mikami E., Kumagai H., Ikeda H., Shimasaki Y., Yoshimura M., Cugia P., Piras F., Scorcu M., Kikuchi N. (2020). Association between the ACE I/D Polymorphism and Muscle Injuries in Italian and Japanese Elite Football Players. J. Sports Sci..

[18] Kumagai H., Miyamoto-Mikami E., Hirata K., Kikuchi N., Kamiya N., Hoshikawa S., Zempo H., Naito H., Miyamoto N., Fuku N. (2019). ESR1 Rs2234693 Polymorphism Is Associated with Muscle Injury and Muscle Stiffness. Med. Sci. Sports Exerc..

[19] Montgomery H.E., Clarkson P., Dollery C.M., Prasad K., Losi M.A., Hemingway H., Statters D., Jubb M., Girvain M., Varnava A. (1997). Association of Angiotensin-Converting Enzyme Gene I/D Polymorphism with Change in Left Ventricular Mass in Response to Physical Training. Circulation.

[20] Juffer P., Furrer R., González-Freire M., Santiago C., Verde Z., Serratosa L., Morate F.J., Rubio J.C., Martin M.A., Ruiz J.R. (2009). Genotype Distributions in Top-Level Soccer Players: A Role for ACE?. Int. J. Sports Med..

[21] Chen Y.J., Li L.J., Tang W.L., Song J.Y., Qiu R., Li Q., Xue H., Wright J.M. (2018). First-Line Drugs Inhibiting the Renin Angiotensin System versus Other First-Line Antihypertensive Drug Classes for Hypertension. Cochrane Database Syst. Rev..

[22] Simonyte S., Kuciene R., Medzioniene J., Dulskiene V., Lesauskaite V. (2017). Renin-Angiotensin System Gene Polymorphisms and High Blood Pressure in Lithuanian Children and Adolescents. BMC Med. Genet..

[23] Danser A.H., Schalekamp M.A., Bax W.A., van den Brink A.M., Saxena P.R., Riegger G.A., Schunkert H. (1995). Angiotensin-Converting Enzyme in the Human Heart. Eff. Deletion Inser. Polymorphism. Circ..

[24] Rigat B., Hubert C., Alhenc-Gelas F., Cambien F., Corvol P., Soubrier F. (1990). An Insertion/Deletion Polymorphism in the Angiotensin I-Converting Enzyme Gene Accounting for Half the Variance of Serum Enzyme Levels. J. Clin. Investig..

[25] Sayed-Tabatabaei F.A., Oostra B.A., Isaacs A., van Duijn C.M., Witteman J.C.M. (2006). ACE Polymorphisms. Circ. Res..

[26] Jones A., Woods D.R. (2003). Skeletal Muscle RAS and Exercise Performance. Int. J. Biochem. Cell Biol..

[27] Zhang B., Tanaka H., Shono N., Miura S., Kiyonaga A., Shindo M., Saku K. (2003). The I Allele of the Angiotensin-Converting Enzyme Gene Is Associated with an Increased Percentage of Slow-Twitch Type I Fibers in Human Skeletal Muscle. Clin. Genet..

[28] Ma F., Yang Y., Li X., Zhou F., Gao C., Li M., Gao L. (2013). The Association of Sport Performance with ACE and ACTN3 Genetic Polymorphisms: A Systematic Review and Meta-Analysis. PLoS ONE.

[29] Fluck M., Kramer M., Fitze D., Kasper S., Franchi M., Valdivieso P. (2019). Cellular Aspects of Muscle Specialization Demonstrate Genotype—Phenotype Interaction Effects in Athletes. Front. Physiol..

[30] Vaughan D., Brogioli M., Maier T., White A., Waldron S., Rittweger J., Toigo M., Wettstein J., Laczko E., Flück M. (2016). The Angiotensin Converting Enzyme Insertion/Deletion Polymorphism Modifies Exercise-Induced Muscle Metabolism. PLoS ONE.

[31] Pescatello L.S., Kostek M.A., Gordish-Dressman H., Thompson P.D., Seip R.L., Price T.B., Angelopoulos T.J., Clarkson P.M., Gordon P.M., Moyna N.M. (2006). ACE ID Genotype and the Muscle Strength and Size Response to Unilateral Resistance Training. Med. Sci. Sports Exerc..

[32] Charbonneau D.E., Hanson E.D., Ludlow A.T., Delmonico M.J., Hurley B.F., Roth S.M. (2008). ACE Genotype and the Muscle Hypertrophic and Strength Responses to Strength Training. Med. Sci. Sports Exerc..

[33] Kang H.-J., Kim C.-H., Park D.-S., Choi S.-Y., Lee D.-H., Nam H.-S., Hur J.-G., Woo J.-H. (2012). The Impacts of ACE Activity According to ACE I/D Polymorphisms on Muscular Functions of People Aged 65. Ann. Rehabil. Med..

[34] Baumert P., Lake M.J., Stewart C.E., Drust B., Erskine R.M. (2016). Genetic Variation and Exercise-Induced Muscle Damage: Implications for Athletic Performance, Injury and Ageing. Eur. J. Appl. Physiol..

[35] Yamin C., Amir O., Sagiv M., Attias E., Meckel Y., Eynon N., Sagiv M., Amir R.E. (2007). ACE ID Genotype Affects Blood Creatine Kinase Response to Eccentric Exercise. J. Appl. Physiol..

[36] Del Coso J., Salinero J.J., Lara B., Gallo-Salazar C., Areces F., Herrero D., Puente C. (2020). Polygenic Profile and Exercise-Induced Muscle Damage by a Competitive Half-Ironman. J. Strength Cond. Res..

[37] Del Coso J., Valero M., Salinero J.J., Lara B., Gallo-Salazar C., Areces F. (2017). Optimum Polygenic Profile to Resist Exertional Rhabdomyolysis during a Marathon. PLoS ONE.

[38] Sierra A.P.R., Lima G.H.O., da Silva E.D., de Souza Maciel J.F., Benetti M.P., de Oliveira R.A., de Oliveira Martins P.F., Kiss M.A.P., Ghorayeb N., Newsholme P. (2019). Angiotensin-Converting Enzyme Related-Polymorphisms on Inflammation, Muscle and Myocardial Damage After a Marathon Race. Front. Genet..

[39] Tanisawa K., Wang G., Seto J., Verdouka I., Twycross-Lewis R., Karanikolou A., Tanaka M., Borjesson M., Di Luigi L., Dohi M. (2020). Sport and Exercise Genomics: The FIMS 2019 Consensus Statement Update. Br. J. Sports Med..

[40] Bahr R., Clarsen B., Derman W., Dvorak J., Emery C.A., Finch C.F., Hägglund M., Junge A., Kemp S., Khan K.M. (2020). International Olympic Committee Consensus Statement: Methods for Recording and Reporting of Epidemiological Data on Injury and Illness in Sport 2020 (Including STROBE Extension for Sport Injury and Illness Surveillance (STROBE-SIIS)). Br. J. Sports Med..

[41] Clarsen B., Pluim B.M., Moreno-Pérez V., Bigard X., Blauwet C., Del Coso J., Courel-Ibáñez J., Grimm K., Jones N., Kolman N. (2021). Methods for Epidemiological Studies in Competitive Cycling: An Extension of the IOC Consensus Statement on Methods for Recording and Reporting of Epidemiological Data on Injury and Illness in Sport 2020. Br. J. Sports Med..

[42] Chiu Y.-H., Lai J.-I., Tseng C.-Y., Wang S.-H., Li L.-H., Kao W.-F., How C.-K., Chang W.-H., Hsieh C.-Y. (2019). Impact of Angiotension I Converting Enzyme Gene I/D Polymorphism on Running Performance, Lipid, and Biochemical Parameters in Ultra-Marathoners. Medicine.

[43] Rigat B., Hubert C., Corvol P., Soubrier R. (1992). PCR Detection of the Insertion/Deletion Polymorphism of the Human Angiotensin Converting Enzyme Gene (DCP1) (Dipeptidyl Carboxypeptidase 1). Nucleic Acids Res..

[44] Lim T., Santiago C., Pareja-Galeano H., Iturriaga T., Sosa-Pedreschi A., Fuku N., Pérez-Ruiz M., Yvert T. (2021). Genetic Variations Associated with Non-Contact Muscle Injuries in Sport: A Systematic Review. Scand. J. Med. Sci. Sports.

[45] Larruskain J., Celorrio D., Barrio I., Odriozola A., Gil S.M., Fernandez-Lopez J.R., Nozal R., Ortuzar I., Lekue J.A., Aznar J.M. (2018). Genetic Variants and Hamstring Injury in Soccer: An Association and Validation Study. Med. Sci. Sports Exerc..

[46] Varillas-Delgado D., Gutierrez-Hellín J., Maestro A. (2023). Genetic Profile in Genes Associated with Sports Injuries in Elite Endurance Athletes. Int. J. Sports Med..

[47] Onori M.E., Pasqualetti M., Moretti G., Canu G., De Paolis G., Baroni S., Minucci A., Galvani C., Urbani A. (2022). Genetics and Sport Injuries: New Perspectives for Athletic Excellence in an Italian Court of Rugby Union Players. Genes.

[48] de Almeida K.Y., Cetolin T., Marrero A.R., Aguiar Junior A.S., Mohr P., Kikuchi N. (2022). A Pilot Study on the Prediction of Non-Contact Muscle Injuries Based on ACTN3 R577X and ACE I/D Polymorphisms in Professional Soccer Athletes. Genes.

[49] Moreno-Perez V., Campos-Vazquez M.A., Toscano J., Sotos-Martinez V.J., López-Del Campo R., Resta R., Del Coso J. (2022). Influence of the Weekly and Match-Play Load on Muscle Injury in Professional Football Players. Int. J. Sports Med..

[50] Tanabe Y., Fujii N., Suzuki K. (2021). Dietary Supplementation for Attenuating Exercise-Induced Muscle Damage and Delayed-Onset Muscle Soreness in Humans. Nutrients.

[51] Massidda M., Scorcu M., Calo C. (2014). New Genetic Model for Predicting Phenotype Traits in Sports. Int. J. Sports Physiol. Perform..

[52] Hughes D.C., Day S.H., Ahmetov I.I., Williams A.G. (2011). Genetics of Muscle Strength and Power: Polygenic Profile Similarity Limits Skeletal Muscle Performance. J. Sports Sci..

[53] Williams A.G., Folland J.P. (2008). Similarity of Polygenic Profiles Limits the Potential for Elite Human Physical Performance. J. Physiol..

